# Enumeration of Sex Workers in the Central Business District of Nairobi, Kenya

**DOI:** 10.1371/journal.pone.0054354

**Published:** 2013-01-25

**Authors:** Joshua Kimani, Lyle R. McKinnon, Charles Wachihi, Judith Kusimba, Gloria Gakii, Sarah Birir, Mercy Muthui, Anthony Kariri, Festus K. Muriuki, Nicholas Muraguri, Helgar Musyoki, T. Blake Ball, Rupert Kaul, Lawrence Gelmon

**Affiliations:** 1 Kenya AIDS Control Project, Universities of Manitoba/Nairobi, Nairobi, Kenya; 2 Medical Microbiology, University of Nairobi, Nairobi, Kenya; 3 Medicine, University of Toronto, Toronto, Canada; 4 Global AIDS Program, CDC, Nairobi, Kenya; 5 National AIDS & STI Control Programme (NASCOP), Ministry of Public Health and Sanitation, Nairobi, Kenya; 6 Public Health Agency of Canada, Winnipeg, Canada; Tulane University, United States of America

## Abstract

Accurate program planning for populations most at risk for HIV/STI acquisition requires knowledge of the size and location where these populations can best be reached. To obtain this information for sex workers operating at 137 hotspots in the central business district (CBD) in Nairobi, Kenya, we utilized a combined mapping and capture-recapture enumeration exercise. The majority of identified hotspots in this study were bars. Based on this exercise, we estimate that 6,904 male and female sex workers (95% confidence intervals, 6690 and 7118) were working nightly in the Nairobi CBD in April 2009. Wide ranges of captures per spot were obtained, suggesting that relatively few hot spots (18%) contain a relatively high proportion of the area's sex workers (65%). We provide geographic data including relatively short distances from hotspots to our dedicated sex worker outreach program in the CBD (mean<1 km), and clustering of hotspots within a relatively small area. Given the size covered and areas where sex work is likely taking place in Nairobi, the estimate is several times lower than what would be obtained if the entire metropolitan area was enumerated. These results have important practical and policy implications for enhancing HIV/STI prevention efforts.

## Introduction

Sex workers (SW) in many settings engage in high-risk behaviour that predisposes them to infection by HIV and other STIs. This group remains a high-risk core group for contracting and transmitting HIV and other STIs, partly because of inconsistent and occasionally incorrect condom use with regular and casual partners [1], [2], [3], [4]. HIV transmission to clients can lead to further infections of clients' partners in the general population [5], [6]. Therefore, sex workers remain among the most important focal points for effective HIV prevention in the general population [7], [8].

Accumulated evidence suggests that the risk of HIV transmission in SW can be reduced through improved STI screening and treatment services, provision of health education, condom demonstration and promotion, HIV counseling, testing, and treatment [9], [10], [11], [12], [13]. However, designing targeted interventions for SW as part of an expanded and comprehensive response to HIV/AIDS control requires reliable population size estimates. These estimates are critical in planning, resource allocation, and monitoring and evaluation that are essential to achieving program impact [8].

The capture-recapture method is an epidemiologic tool for estimating population size from two independent, overlapping sources. The methodology originates from wildlife biology and demography, and has been adapted in epidemiology to provide population parameter estimates based on two or more incomplete sources; to refine incidence estimates and their upper and lower bounds; and to estimate the completeness of apparently exhaustive surveys [14],[15].

Since incomplete data exists on the population size of sex workers in Kenya, an enumeration was conducted within the Nairobi central business district (CBD) in April 2009 as a pilot project. This enumeration was made possible by a grant from the Centre for Disease Control-U.S. President's Emergency Plan for AIDS Relief (CDC-PEPFAR).

## Methods

### Ethical approval

The protocol was approved by the Kenyatta National Hospital/University of Nairobi Ethics Review Committee (KNH/UNO-ERC) and the University of Manitoba Research ethics board. During the enumeration exercise, SW peer leaders helped identify SW at all hot spots; these individuals were then approached by enumerators, who explained the purpose of the study. Verbal consent was obtained from SW who admitted to engaging in sex work. It was not feasible to obtain written consent, given that duration of contact with SW was very short, in assorted environments (noisy bar, disco, poorly lit streets, etc.), and because SW is illegal in Kenya.

### Geographical area

For the purposes of this enumeration, the Nairobi CBD was divided geographically into three sections, the whole area defined based on boundaries of Uhuru Highway to the South, Haile Selasie Avenue and Ring Road to the East, Ngara and Muranga Road, and Globe roundabout to the North, and Slip Road and University Way to the West. All sex work hot spots within this area and along these roads were included in the enumeration exercise. Individuals were counted if they were self-reported to be engaged in sex work on the two days of enumeration in the CBD, regardless of sex and age. The project was conducted in two parts: hotspot mapping and enumeration exercise utilizing a capture-recapture methodology [16].

### Mapping, pre-testing, and enumeration

Over a 5-day period, peer educators prepared a list of all known hotspots within Nairobi CBD. The research team then interviewed selected key informants including taxi drivers, bar owners, and bouncers to identify and confirm potential hot spots. Validation of hot spots, particularly those named by more than one informant, was confirmed by physical visits by a research team member and peer leader. The sex worker peer leaders were selected based on their experience and knowledge of the defined geographical area. The final list of validated SW hotspots within the designated CBD area were then mapped using Global Positioning System (GPS) devices by teams consisting of research assistants, peer leaders, and personnel competent in operation of GPS devices. Locations were uploaded into Google Earth Plus. Codes were assigned to the hotspots based on the sections of the CBD map obtained from the Survey of Kenya.

Groups of SW peer leaders and research assistant pretested the study tools for practicality and applicability in the weeks prior to the enumeration, with feedback used to modify the tool accordingly. A one-day training was held for the enumeration team, focusing on reasons for and objectives of the sex workers enumeration in the CBD, methodology and how to reach out to sex workers at hotspots. Each team was assigned to cover specific hotspots, and practicality of the exercise determined. Problems faced by field staff during the pre-test were raised and discussed.

The timing of the actual enumeration was determined based on consultations with the peer leaders to coincide with peak SW hours (7pm to 3am) and time in the month. During the enumeration, all SW who self-identified as being ready for clients (i.e.“on the market”) through the peers leaders were counted using the capture and re-capture methodology that was conducted two weeks apart. Serialized enumeration cards were printed in duplicate, colour-coded for the two different days of the enumeration, and included information regarding the date, hotspot location, enumeration team, and gender of SW. Those enumerated during round 1 were also asked to keep the cards safely for presentation, if applicable, during the round 2 ‘re-capture’. On both enumerations days, SW who were identified by peer leaders but declined to take cards were marked as “refusals”. Similarly, in cases where peer leaders identified SW who were engaged with clients, they were not disrupted but recorded as “busy”. Two weeks later, all hotspots were re-visited and SW counted and re-issued with enumeration cards. Serialized enumeration cards on day 2 were marked as “capture” or “recapture”. Those who had received cards on the first day were recorded as “recaptures” and asked to return cards from day 1. The enumeration cards also provided details where sex workers could access free HIV/STD services.

### Calculation of population size

Data was entered Microsoft Access and analyzed in SPPS version 17. The sizes of captures and re-captures were used to calculate the population size.

The formula is as follows:

N = (number in first capture×number in second capture)/number in both captures.

95% confidence interval was calculated using the following formula:

95% C1 = N±1.96 √Var (N), where Var (N) is calculated as follows: Var (N) = [(first capture×second capture) (first capture-recaptures) (second capture-recaptures)]/[(recapture)] [3], [14].

## Results

In the mapping exercise, 137 hotspots were identified, and included 71 bars (51.8%), 47 street-based venues (34.3%), 10 hotels (7.3%), 5 sex dens (3.6%), and 4 (2.9%) strip clubs. Eleven (9%) of these hotspots were noted to be busy during both day and night and therefore the timing of the enumeration was expanded for these sites. A total of 34 teams, each consisting of at least two enumerators and one or more sex workers peer leaders, were formed. The teams were further clustered into five groups each under two supervisors. The supervisors oversaw how the group members conducted the enumeration at allocated hotspots.

On day one, a total of 3,070 sex workers were counted, 327 sex workers refused, and 228 were busy with clients (Table 1). On day two, 2,901 sex workers were captured, 1,290 of whom were recaptures. On day 2, 243 sex workers refused to participate in the exercise while another 205 were busy with clients. Based on these data, Nairobi CBD was estimated to host 6,904 sex workers with 95% confidence intervals between 6,690 and 7,118. On the first day of the enumeration, five percent of the total sex workers population captured were male, and the second day, males represented two percent of the population.

**Table 1:** Breakdown of SW enumerated at each type of hot spot.

	*No. of Spots*	*Capture*	*% of Total Capture*	*Recapture*	*Busy* *	*Refused* *
Bar	71	1811	59.0	709	186	157
Hotel	10	457	14.9	194	50	16
Sex Den	5	37	1.2	22	4	2
Street	47	725	23.6	334	84	44
Strip Club	4	40	1.3	31	3	9
**TOTAL**	**137**	**3070**	**100**	**1290**	**327**	**228**

* Day 1 of enumeration.

The majority of SW enumerated in the Nairobi CBD operated in bars (59.0% of those captured on day 1), followed by the street-based venues (23.6%, Table 1). Hotel-based SW represented 14.9% of the overall population, while sex den and strip club-based SW, likely based on the small numbers of these types of hot spots included, represented the remainder. We also categorized hot spots, depending on the volume of SW counted at each. These data suggest that a relatively small proportion of hot spots (18.3%) contributed 65.2% (n = 2,913) of SW that were enumerated (Table 2). This could have important implications in terms of where to focus mobilization and intervention efforts to reach the highest percentage of the SW population in Nairobi CBD.

**Table 2:** Distrubution of SW enumerated based on SW volume at each hot spot.

*SW Captured/Spot (Day 1)*	*No. of Hot Spots (% of total)*	*Total SW Captured at these Spots*
Less than 10	56 (40.9)	144
10–49	57 (41.6)	923
50–99	16 (11.7)	736
More than 100	9 (6.6)	1267
**TOTAL**	**137**	**3070**

The distance of the location of hot spots with most sex workers (>50 captured in round 1) from our SWOP City clinic is less than one kilometer (Table 3, mean 990 m, range 460–2,000 m). The location of this clinic should be advantageous, allowing participants to access HIV/STI prevention and care services in close proximity to locations where they conduct business.

**Table 3:** Distance of selected hot spots from the SWOP City clinic.

*Location code*	*Location type*	*Distance from SWOP (m)*
A014	Hotel	950
A031	Bar	850
A034	Bar	1390
B010	Bar	970
B015	Hotel	900
B017	Bar	730
B021	Bar	820
B024	Bar	900
B025	Bar	2000
B030	Bar	560
B031	Bar	460
B034	Bar	620
B035	Bar	725
C001	Bar	1340
C005	Bar	1100
C008	Bar	1500
C020	Bar	850
C028	Bar	1150
**n = 18**	**AVERAGE DISTANCE**	**990**

## Discussion

In this enumeration, a two-sample capture-recapture calculation was used to estimate that 6,904 sex workers were operating in the Nairobi CBD in April 2009. This is among the first robust enumerations of this important most-at-risk population (MARP) in Nairobi, which has important implications for planning purposes. Moreover, geographic data demonstrate close proximity between our sex worker outreach program and sex work hot spots, which themselves cluster in a relatively small area. Location of HIV/STI prevention and care services near sex workers' places of business is likely an important variable for them to access these services regularly, as has been shown for MARPs on long-distance truck routes in East Africa [17].

Two samples of sex workers operating at 137 mapped hotspots were drawn during peak sex work; considering the large number of enumerators, and the size of teams ranging from three to five members, assigned at most three hotspots that were adjacent to each other, we predict that hotspot coverage was adequate. This arrangement aimed to minimize the chances of missing the target population working in the CBD at the time of enumeration. Furthermore, we have scaled up this exercise for a recent World Bank-funded initiative to enumerate sex workers across Kenya, and found this to be very feasible, even at a large scale, at relatively low cost (unpublished).

For capture-recapture estimates to be valid, certain conditions need to be met: the target population must be closed; the two samples must be independent; recaptures must be correctly identified; the probability of being captured during both rounds equal; and that the people captured must belong to the target population. Although the target population in this study is mobile, little change was anticipated given the short interval between samples. The sample locations did not change, and therefore the samples were independent. Ensuring correct identification of recaptures prevented overstatement of the actual number of recaptures that would have led to an underestimate of the target population. SW counted the first day may have been more likely to be included on the second day, due to familiarity with enumerators. An increase in the recaptures and subsequently a reduction of the estimate may have been balanced by SW who refused or were too busy to receive enumeration cards on the first day, but included on the second day. Double counting on both days was minimized by not giving incentives that would have facilitated movements of SW from one hotspot to another, and by establishing whether a card had been received from another enumerator that evening. Even though peer leaders ensured that the people counted were SW, a few individuals might have received the enumeration cards by mistake, possibly because they expected to gain in return.

**Figure 1:**
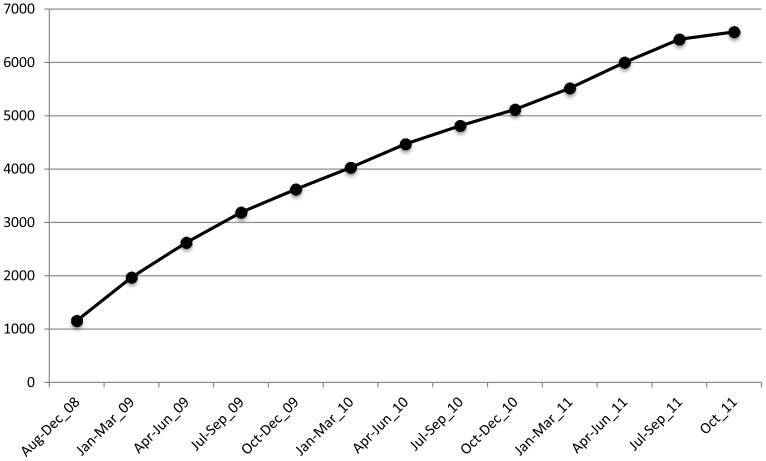
Cumulative enrollment of female sex workers at the SWOP-City Clinic in the Nairobi Central Business District, Aug 2008 until Oct 2011.

By the end of 2011, we recruited and enrolled 6,572 female sex workers to the SWOP-City clinic, in the same area where this enumeration took place (Fig. 1). Based on this enumeration in 2009, it appears nearly 95% of sex workers in this area are being reached. However, this clinic draws attendees from many areas of Nairobi. SW who access this clinic receive free education and risk reduction counseling, condoms, and HIV/STI testing and care. Therefore, although coverage of the Nairobi CBD is dramatically improving, it is likely that there are still more sex workers who need to be reached. Furthermore follow-up of enrolled SW is important to maximize the impact of HIV/STI prevention messages. To ensure access to STI/HIV services has been improved for sex workers in Nairobi County, six dedicated clinics were opened in 2011.

The current study did not use any male sex worker peer leaders to identify male SW. This probably explains why only five percent of the total estimated SW population is male, despite belief that there are many male sex workers in the CBD. This also implies that even though reaching out to SW is generally difficult, male SW may be even more difficult to reach. Through a more targeted approach, we have enrolled 463 male SW in SWOP-City clinic by the end of 2011. The proportion of the actual Nairobi CBD male SW population this represents is difficult to ascertain. Another limitation is that sex workers are a mobile population, and the methodologies used here might not capture this challenging aspect of population estimation. Future studies should devise better strategies to take this important (and potential risk) factor into account.

Finally, it should also be appreciated from a map of Nairobi that the area covered represents a small proportion of the total metropolitan area, and that there are at least half a dozen other districts around town that contain a large number of entertainment bars and clubs, or where sex workers are known to work in large numbers. Indeed, preliminary data from an ongoing mapping exercise suggests that the total number of SW in greater Nairobi is several times that in the CBD. However, the methodology utilized for this study seemed to work well, and could be easily replicated in other locations in Nairobi or elsewhere. There is a need to accurately estimate the size and location of this high-risk population since it has important implications for the design of successful HIV/STI prevention programs.
