# Correction: Enumeration of Sex Workers in the Central Business District of Nairobi, Kenya

**DOI:** 10.1371/annotation/29718092-c94c-4c98-b5a8-253a6252493a

**Published:** 2013-11-20

**Authors:** Joshua Kimani, Lyle R. McKinnon, Charles Wachihi, Judith Kusimba, Gloria Gakii, Sarah Birir, Mercy Muthui, Anthony Kariri, Festus K. Muriuki, Nicholas Muraguri, Helgar Musyoki, T. Blake Ball, Rupert Kaul, Lawrence Gelmon

After the publication of this article, concerns were raised about the inclusion of a figure which reported a map of the Nairobi CBD showing the location of the sex worker hot spots involved in the study and the number captured at each location during the enumeration.

In light of the possible implications that the public availability of this information can have on the Nairobi sex workers, the authors have decided to withdraw this map from the publication. The article has therefore been republished without the original Figure 1. Any readers with an academic interest in the information originally displayed in the figure may contact the authors directly in relation to this information. 

